# Examining the Determinants of COVID-19 Severity: A Cohort Study in Morocco of 915 Patients

**DOI:** 10.7759/cureus.32914

**Published:** 2022-12-25

**Authors:** Zaynab Mahdi, Faïza Charif, Adil Gourinda, Karima Sammoud, Fadila Bousgheiri, Hassana Belafki, Fadila Salmane, Wiam Ftouh, Mariem Benkacem, Adil Najdi

**Affiliations:** 1 Laboratory of Epidemiology and Public Health, Mohammed VI University Hospital of Tangier, Tangier, MAR; 2 Endocrinology, Diabetes and Metabolic Diseases, Mohammed VI University Hospital of Tangier, Tangier, MAR

**Keywords:** covid 19, clinical outcomes, sars-cov-2 infection, prognosis, severity, risk factors

## Abstract

Introduction: Coronavirus disease 2019 (COVID-19) is unpredictable and it varies from mild to severe and critical forms that are associated with a higher mortality rate. Risk factors associated with severe forms of severe acute respiratory syndrome coronavirus 2 (SARS-CoV-2) infection have been investigated worldwide. We aimed to evaluate the clinical course of severe COVID-19 patients and to compare them with the non-severe patients concerning clinical and epidemiological characteristics, biological parameters and outcomes and thus, highlight the factors associated with severe forms of COVID-19 in our country.

Methods: This is a single-center, ambidirectional cohort study, conducted in Tangier’s COVID-19 care premises. We included diagnosed COVID-19 patients between August 2020 and October 2021. Sampling was performed through stratification according to clinical forms. All patients were followed-up throughout disease evolution, until remission for mild to moderate forms and 30 days after discharge for hospitalized patient’s group (severe to critical forms). Data were collected using the WHO International Severe Acute Respiratory and Emerging Infection (ISARIC) case report form (CRF) and extracted from medical records alongside with interviews with patients and their relatives.

Results: Among 915 included COVID-19 patients in Tangier, the non-severe group comprised 344 (37.6%) patients and the severe group comprised 571 (62.4%) patients. Some 514 were males (56.2%) and 401 were females (43.8%) and the mean age was 56.01 years (±16.76). The mean delay from onset of symptoms to diagnosis was 6.65 days ±4.68 in the severe group and 5.4 days ±4.57 in the non-severe group (p<0.001). Among the severe patient’s group, 230 (40.3%) patients were admitted to the resuscitation unit, 258 (45.2%) patients were deceased during hospitalization, 313 (54.8%) were discharged alive, and 16 deaths occurred after discharge. Demographic, clinical, and biological characteristics showed significant differences between non-severe group and severe group. Multivariable logistic regression analysis showed increased odds of severity with male gender (adjusted odds ratio, aOR=2.91, p<0.003), age over 65 years old (aOR=2.68, p<0.001), diabetes (aOR=2.18, p<0.03), elevated D-dimers (>1 mg/mL) (aOR=6.09, p<0.001), superinfection (aOR=3.78, p<0.001), and baseline lymphopenia < 1000c/mm3 (aOR=8.66, p<0.001).

Conclusion: The high-risk factors for developing severe COVID-19 are age > 65 years, male gender, diabetes, elevated D-dimers, baseline lymphopenia, and superinfection. To predict severe and fatal COVID-19, factors identified may be used in the development of prediction tools for COVID-19 prognosis and risk stratification. Recalling the importance of considering at-risk populations, the management of epidemics must be planned in conjunction with the specificity of each community. Findings from our study may serve for health economic analyses and research in order to assist public health decisions in the future and should be integrated into health emergency preparedness and response strategies ensuring a resilient health system.

## Introduction

Severe acute respiratory syndrome coronavirus 2 (SARS-CoV-2) infection’s clinical spectrum is very wide, ranging from complete well-being to severe life-threatening viral pneumonia along with acute respiratory distress syndrome (ARDS) and multiple other organs complications [1]. Severe forms of coronavirus disease 2019 (COVID-19) disease consume care services and occupy intensive care beds, which constitutes an economic burden that stresses any health system, in addition to the high human cost of the disease in severe forms where the mortality rate is higher.

In Morocco, the epidemic was first declared in March 2020 and as of January 2021, the time frame in which data collection began, the total number of COVID‑19 cases in Morocco had reached 462 542, with a recovery rate of 94.7%, a case fatality rate of 1.7%, and a resuscitation bed occupancy rate of 27.4% [2], compared to the 2021 worst wave that occurred in our country, the total number of COVID‑19 cases in Morocco had reached 860 948 by the end of august 2021, with a recovery rate of 92.2%, a case fatality rate of 1.5%, and a resuscitation bed occupancy rate of 45.8% [2]. And as of September 2022, Morocco succeeded in vaccinating 23 397 476 of the total population with two doses [2] with a vaccination coverage rate around 64%. During that same period, the total number of COVID‑19 cases reached 1 264 822, with a recovery rate of 98.7%, a case fatality rate of 1.3%, and a resuscitation bed occupancy rate of 0.1% [2]. And thankfully, the recent decrease in daily cases implies that the spread of the pandemic is being effectively controlled and that clinical and epidemiological research on COVID-19 are allowing the enhancement of the COVID-19 disease management through learned societies recommendations. This improvement is also justified by the advent of vaccination but it is now widely acknowledged that the risk of mutations is constant, emerging different variants of SARS-CoV-2 and consequently, making the pandemic risk a permanent threat.

The main risk factors associated with severity might be classified into factors related to health-care access encompassing geographical inequalities in access to care, delay in care and treatment [3] and other risk factors including constitutional factors; age over 65, male gender [4-6], comorbidities as asthma, chronic lung disease, history of tuberculosis, hypertension, diabetes, cardiovascular disease, cancers, immunodepression, liver disease, and renal failure [4-5, 7]; individual behavioral factors (lifestyle); such as obesity, smoking, vitamin D deficiency [5, 8], and drug intake like corticosteroid therapy and non-steroidal anti-inflammatory drugs (NSAIDs) [7]. And although several researches have investigated COVID-19 patients epidemiological and clinical specifications worldwide, producing fast and significant findings, there remains a certain lack of data concerning our context and evidence on factors related to disease severity and other potential factors that are yet to be explored, as severe forms and deaths from COVID-19 have occurred among individuals without known risk factors; this raises a number of questions about this unpredictable evolution and other uncontrollable risk factors are still unknown. It is, therefore, essential to accurately identify avoidable determinants of severe forms of COVID-19 in order to prevent and control them, ensuring a better care and thus a better prognosis.

It should also be noted that when assessing studies from different socioeconomic backgrounds and different geographic locations, it is important to consider that each context retains its specificities and we did observe an important North-South gradient of morbidity and mortality [3]. Morocco belongs to a southern region (Africa) and scientists predicted that low-and middle-income countries, such as those in Africa, would be the most negatively affected. But in contrast to the COVID-19 prediction, Africa has reported the least number of cases and deaths and there is still uncertainty and inadequate research on COVID-19. Africa may have been spared the worst of the pandemic due to its population that is younger and its warmer climates but what other factors could have contributed? This raises concerns and prompts us to think about the particularities of our country, particularly when applying the recommendations and guidelines gathered from the literature.

The aim of this study was to identify the main determinants of severe forms in patients with COVID-19 in Morocco. With the following specific objectives: identifying the groups at risk and describing the management of patients and the therapeutic results, describing hospital stay and delay of treatment intake and finally comparing between non-survivors and survivors in the severe group.

## Materials and methods

Ethical approval, consent, and confidentiality

We obtained Approval for this work through the Ethics Committee of Fez (Approval number: 03/21). We obtained oral informed consent from all patients enrolled in the study and from legal guardians of deceased patients. We anonymized patients’ identities and we committed to personal data protection and confidentiality.

Design

We conducted an ambidirectional cohort study (retrospective and prospective) of patients with COVID-19 disease between 14 August 2020 and 14 October 2021 among a sample of patients with COVID-19 diagnosis at the COVID-19 care premises in Tangier, Morocco.

Subjects

Patients with COVID-19 disease in Tangier were diagnosed during the study period. We included subjects over 18 years old, meeting the Ministry of Health definition of a confirmed COVID-19 case, regardless of clinical form [9]. The inclusion criteria are described as follows: confirmed SARS-CoV-2 infection by molecular examination (reverse transcriptase-polymerase chain reaction, RT-PCR or equivalent test) or rapid antigenic test. And any suspected case with the following: CT images suggestive of COVID-19; and an evocative epidemiologic context (contact with a confirmed case during the infectious period; or link to a cluster; or a healthcare professional working in a healthcare facility or laboratory). Any patient who refused (he/she or his/her legal representative) to participate in the study was not included.

Enrollment Period

The first period was from August 2020 to January 2021 during which we studied already existing medical records of patients meeting the inclusion criteria. During the second period (from February 2021 to October 2021) we switched to the prospective inclusion of newly diagnosed and/or admitted subjects over a nine-month period.

Sample Size

This study is part of a bigger project "Identifying the determinants of severity and mortality in patients with COVID-19 in Morocco." This project aimed to identify COVID-19 severity risk factors and the high-risk groups, describe the management of patients and the therapeutic results, study predictors of mortality among patients with severe and/or critical forms of COVID-19, study the clinical and epidemiological profile of deceased patients, describe patient survival, estimate the lethality of COVID-19, calculate the standardized mortality on the world population, and compare it with other countries.

The sample size was computed according to all project objectives. Sampling was based on stratification on clinical forms with:

N1: mild and moderate forms to be recruited among patients treated at home.

N2: severe and critical forms to be recruited among hospitalized patients with a mortality rate of 15% [10].

Considering this mortality rate and for a minimal number of expected deaths fixed at 60, we estimated N2 sample to be 400 hospitalized subjects. The number of subjects to be included was calculated at 600 subjects (N1+N2) of which 400 were severe and critical forms (N2) and 200 mild and moderate forms (N1). The proportion of N2 in the sample was emphasized so that the minimum required number of deaths could be reached to meet the analytical objective.

Data collection

In the retrospective phase, we used records of patients diagnosed since August 2020, these patients or their families were contacted by phone to complete the lacked information. Then, in the prospective phase, we used records of newly diagnosed patients from February 2021 onwards, who were treated either at home or in hospital facilities (COVID intensive care units and COVID resuscitation units), as well as interviews with patients and their families.

A case report form (CRF) was dedicated to this study. It was developed using the modified International Severe Acute Respiratory and Emerging Infection (ISARIC) CRF; established by the WHO to standardize the data collected, which will allow comparability with other populations [11].

This CRF consists of four modules: Module 1: to be completed at the first consultation or first day of hospitalization; Module 2: to be completed at D7, D14, D21, and D28 of hospitalization/follow-up. This module consists of two parts: the first part is for inpatients and the second for patients treated at home; Module 3: to be completed at discharge from hospital, or after death; Module 4: to be completed for patients discharged alive, at D15 and D30 after discharge.

Variables

The primary outcome of this study was severity and the secondary outcome was in-hospital mortality. To define the primary outcome, we used the Clinical Case Classification Criteria: Asymptomatic case: No clinical manifestations; Mild case: Symptoms suggestive of COVID-19 without signs of pneumonia; Moderate case: Pneumonia, without signs of severity; or Mild case with one or more risk factors; Severe case: Signs of severity requiring intensive care hospitalization without respiratory support and Critical: Need for respiratory assistance (invasive or non-invasive).

In our statistical analysis the main variable was defined as follows: Severe and critical: patients who meet the definition of severe or critical COVID-19 forms at the admission or during the follow-up period. Non-severe: patients with no symptoms, mild or moderate COVID-19 forms either at the admission or during the follow-up period. For the secondary outcome, severe cases were classified according to their survival status; patients discharged alive and remained alive 30 days after discharge were considered survivors. And patients who died in hospital or in the 30 days after discharge were considered non-survivors.

To identify biological abnormalities, we used the thresholds listed in Annex 1, we extrapolated these thresholds from published cohort data and from reference values used at our hospital laboratory. To identify the biological inflammatory syndrome, we have used the following inflammatory markers: C reactive protein, white blood cells count, erythrocyte sedimentation rate, ferritin, and procalcitonin. To identify a mildly elevated liver enzyme we used the threshold listed in annex for both aspartate transaminase and alanine transaminase. And to identify an acute renal failure we used the threshold listed for elevated creatinine and urea.

For each patient, we collected sociodemographic data (age, sex, marital status, occupation, socioeconomic level, place of residence); date of admission, hospitalization department (admission unit: intermediate care unit, intensive care unit and resuscitation unit), personal and medical history: we searched for diabetes, hypertension, chronic respiratory failure, stroke, chronic obstructive pulmonary disease (COPD), obesity, dysthyroidism, psychiatric disorder, chronic liver disease, chronic kidney disease, chronic dialysis, smoking, long-term drug use [angiotensin converting enzyme (ACE) inhibitors, angiotensin II receptor blockers (ARBs), nonsteroidal anti-inflammatory drugs (NSAIDs), statins, antiplatelet agents, oral steroids, immunosuppressive agents, antivirals, antibiotics, anticoagulants, and other medications) and vaccination against COVID-19. On admission, we collected Clinical Case Classification, symptoms, date of onset of symptoms, respiratory symptoms and non-respiratory symptoms, physical signs on clinical examination and vital parameters (weight, height, BMI, temperature, SaO2, heart rate, respiratory rate, blood pressure, assessment of the general condition, performance status, etc). Upon evolution, we collected clinical recovery or worsening data, treatments received, side effects and how they were managed, resuscitation stay and its duration and death. We also collected result of diagnostic RT-PCR or rapid antigen test, biological parameters at admission and initial imaging [initial chest CT with extent of lesions: extent of lung involvement: grade 1 for minimal involvement (<10% extent of lung involvement), grade 2 for moderate involvement (<10%-25% extent of lung involvement), grade 3 for extensive involvement (25%-50% extent of lung involvement), grade 4 for severe involvement (50%-75% extent of lung involvement), and grade 5 for critical involvement (>75% extent of lung involvement); other CT abnormalities, CO-RADS score and ECG results] we also collected radiological worsening data upon evolution, (thoracic CT, Angioscanner), and follow-up parameters.

Patient inclusion and follow-up

The hospitalization, treatment, care, and discharge of the patients were decided in accordance with the guidelines of the Moroccan Ministry of Health [12].

**Figure 1:**
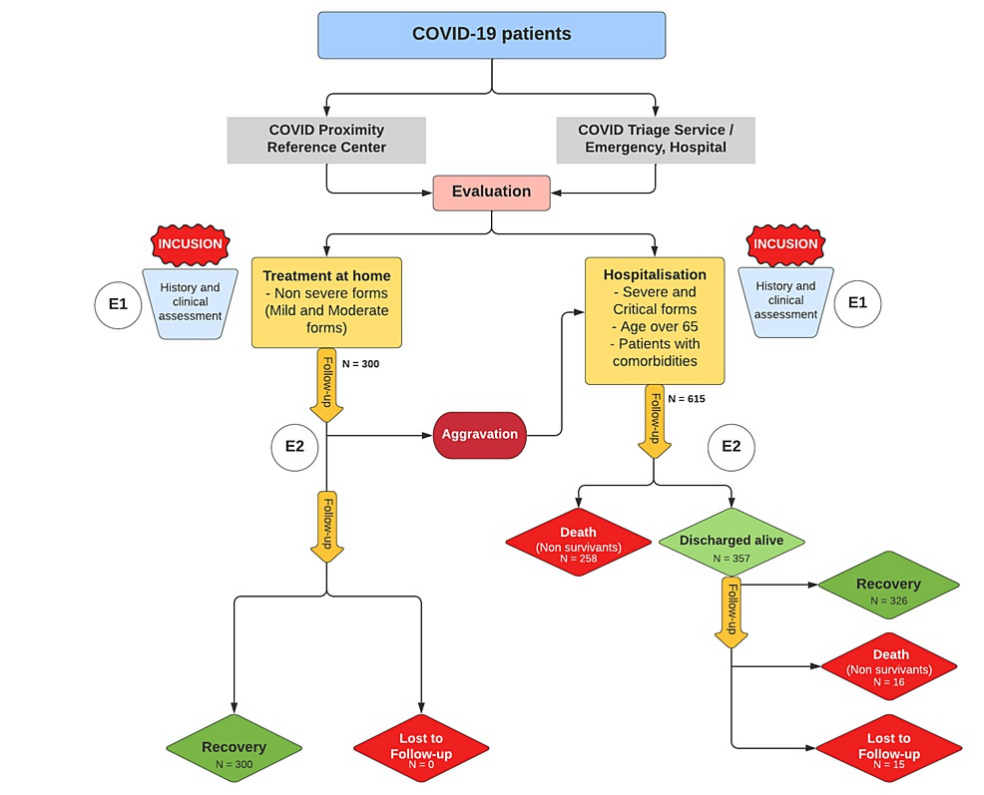
Flowchart of patients from inclusion to end of follow-up. E1: Admission data; E2: Follow-up data

After initial evaluation, patients were classified into two groups according to clinical assessment, age, and potential comorbidities (Figure 1).

Asymptomatic, mild, and moderate cases were treated on an outpatient basis and followed-up using phone calls or at the COVID-19 proximity reference center when needed, during 30 days after inclusion.

Severe and critical cases, cases with comorbidities and patient over 65 of age were all systematically admitted in hospital thus the follow-up was made daily during all the inpatient period as well as 30 days after discharge using phone calls.

The following flowchart explains the inclusion and follow-up procedure:

Statistical analysis

The data collected were entered and analyzed using SPSS software (IBM SPSS Statistics Version 21, IBM Corp., Armonk, NY). A descriptive analysis was performed, for quantitative variables we calculated the means and for qualitative variables, we calculated the percentages. A univariate analysis was then conducted, we compared means using the Student's t test or ANOVA, and percentages were compared using the Pearson Chi-square test or Fisher’s exact test. A multivariate analysis was performed by a logistic regression model adjusting for potential confounders. In all analysis, an estimate was considered statistically significant if the two-sided p-value was below the pre-selected threshold of 0.05.

Ethical approval, consent, and confidentiality

We obtained approval for this work through the Ethics Committee of Fez (Approval number: 03/21). We obtained oral informed consent from all patients enrolled in the study and from legal guardians of deceased patients. We anonymized patients’ identities and we committed to personal data protection and confidentiality.

## Results

Demographics and baseline characteristics

Our study included 915 confirmed COVID-19 cases. The non-severe group comprised 344 patients (37.6%) and the severe group 571 (62.4%). The sample consisted of 514 males (56.2%) and 401 females (43.8%) with a sex ratio of 1.28. In the severe-critical group, 360 were males (63.0%) and 211 were females (37.0%), the sex ratio was 1.71. And in the non-severe group, 154 (44.8%) were males and 190 (55.2%) were females, the sex ratio was 0.81. Of the 401 female patients, nine were pregnant (2.2%).

The mean age of all patients was 56.01 years (±16.76). Diabetes (37.4%), high blood pressure (26.7%) and cardiovascular disease (8.3%) were the most common co-existing conditions; 45 patients had chronic lung disease (5.0%), 37 had chronic kidney failure (4.1%), 34 had asthma (3.7%), 33 had dyslipidemia (3.7%), 16 had cancer (1.8%), 9 suffered from tuberculosis (1.0%), one patient had HIV, and one patient had chronic liver failure. As for the vaccination status, 101 (11.3%) patients (n=896) were vaccinated, of whom 75 (74.3%) had received two doses and 26 (25.7%) had received only one dose.

Clinical outcomes

Most patients with confirmed COVID-19 had fever and respiratory symptoms; 622 (68.0%) patients had fever, 551 (61.0%) had dyspnea, and 611 (37.7%) presented with cough. Other symptoms included headache (45.3%), chest pain (26.3%), myalgia (25.2%), diarrhea (22.4%), and nausea or vomiting (20.0%).

The mean delay from symptom onset to diagnosis was 6.65 days ±4.68 in the severe group and 5.4 days ±4.57 in the non-severe group. And for pre-admission medication, the use of chronic medication or any use of medication during the last 14 days before admission were investigated, 48 (8.4%) of severe group patients were on ACE inhibitors vs 11 (3.2%) from the non-severe group, 67 (11.7%) on angiotensin II receptor blockers (ARBs) vs 22 (6.4%), 36 (6.3%) on steroids vs 22 (6.4%), 35 on statins (6.1%) vs 11 (3.2%), 52 (9.1%) on antiplatelets vs 10 (2.9%) and 62 (10.9%) on anticoagulants vs 9 (2.6%).

Initial CT scans showed minimal to moderate lung involvement (<25%) in 90 patients (13.8%), extensive involvement (25%-50%) in 192 patients (29.4%), severe involvement (50%-75%) in 225 patients (34.5%), and critical involvement (>75%) in 146 patients (22.4%).

Patients received treatment according to the protocol of the ministry of health, 554 (62.1%) received the combination of azithromycin and hydroxychloroquine, 208 patients (32.2%) received azithromycin alone, 23 (2.6%) received hydroxychloroquine alone, and 107 (12.0%) received neither hydroxychloroquine nor azithromycin, 74.3% received vitamin D, and 86.5% received zinc. In the severe group, 530 (77.5%) of patients received LMWH (low molecular weight heparin) vs 154 (22.5%) in the non-severe group, 314 (511%) vs 18 (6.0%) received steroids and 356 (80.4%) vs 87 (19.6%) received additional antibiotics (besides azithromycin).

Table 1 shows patients’ baseline characteristics including demographics, clinical characteristics, and comorbidities along with delays from symptoms onset to diagnosis and admissions in both severe and non-severe groups.

**Table 1:** Epidemiological and clinical features of COVID-19 patients according to severity. *Values represented in this table are numbers (frequencies %) except where it is indicated otherwise. *HBP, high blood pressure; BMI, body mass index; SBP, systolic blood pressure; DBP, diastolic blood pressure; SpO2, oxygen saturation; bRR, brut relative risk *p values state differences between non-severe and severe patients *The frequencies listed in this table represent the proportion of patients with each finding among those tested or assessed for the finding. Not all patients were tested or assessed for each.

COVID-19 patients				
Characteristics	Non-severe (n=344)	Severe (n=571)	p-value	_b_RR
Age subgroups (n=915)				
18-49 years	149 (82.8%)	31 (17.2%)	<0.001	
40-64 years	156 (37.6%)	259 (62.4%)		
>65 years	39 (12.2%)	281 (87.8%)		
Gender (n=915)				
Women	190 (47.4%)	211 (52.6%)	<0.001	1
Men	154 (30.0%)	360 (70.0%)		2.11
Socioeconomic status (n=741)			<0.001	
High	54 (65.9%)	28 (34.1%)		1
Medium	161 (44.7%)	199 (55.3%)		1.62
Low	110 (36.8%)	189 (63.2%)		1.85
Admission of a family member to the resuscitation unit (n=777)	27 (50.0%)	27 (50.0%)	0.238	0.72
COVID-related death in the family (n=782)	24 (46.2%)	28 (53.8%)	0.537	0.84
BMI (kg/m2) (n=597)			0.085	
Normal weight	121 (59.6%)	82 (40.4%)		1
Overweight	114 (52.1%)	105 (47.9%)		1.19
Obesity	85 (48.6%)	90 (51.4%)		1.27
Comorbidities				
HBP (n=906)	48 (19.8%)	194 (80.2%)	<0.001	3.25
Diabetes (n=915)	50 (14.6%)	292 (85.4%)	<0.001	6.15
Cardiovascular disease (n=905)	15 (20.0%)	60 (80.0%)	0.001	2.63
Chronic lung disease (n=908)	16 (35.6%)	29 (64.4%)	0.741	
Asthma (n=909)	14 (41.2%)	20 (58.8%)	0.683	
Chronic renal failure (n=907)	5 (13.5%)	32 (86.5%)	0.002	4.09
Cancer (n=908)	3 (18.0%)	13 (81.3%)	0.110	
Smoking (n=822)	23 (44.2%)	29 (55.8%)	0.386	
Chronic medication				
ACE inhibitors (ACEIs) (n=902)	11 (18.6%)	48 (81.4%)	0.001	2.85
Angiotensin II receptor blockers (ARBs) (n=902)	22 (24.7%)	67 (75.3%)	0.006	2.00
Vital parameters [mean(±SD)]				
Temperature (n=584)	36.8 (±0.68)	37.2 (±3.31)	0.295	
Heart rate (n=859)	82.86 (±15.80)	96.48 (±17.08)	<0.001	
Respiratory rate (n=465)	25.64 (±13.88)	28.34 (±8.48)	0.095	
SBP (n=572)	131.66 (±27.34)	131.38 (±23.96)	0.938	
DBP (n=572)	77.81 (±19.60)	76.53 (±33.07)	0.791	
SpO2 in ambient air (n=863)	97.81 (±2.68)	77.34 (±14.86)	<0.001	
Lung involvement (n=653)			<0.001	
Minor to moderate <25%	53 (58.9%)	37 (41.1%)		1
Extended 25%-50%	34 (17.7%)	158 (82.3%)		2.00
Severe 50%-70%	1 (0.4%)	224 (99.6%)		2.34
Critical >75%	0	146 (100.0%)		2.43
Symptom - diagnosis window [mean(±SD)] (n=826)	5.40 (±4.57)	6.65 (±4.68)	<0.001	
Diagnosis - admission window [mean(±SD)] (n=915)	1.73 (±2.08)	1.84 (±3.13)	0.565	

Laboratory findings

Laboratory parameters were measured and recorded for all patients on the day of admission and then during evolution. Levels of white blood cells ( /L) had an average of 13.03 (±15.91) in the severe group vs 7.05 (±5.51) in the non-severe group, the cell count of lymphocytes ( /L) was 1.27 (±1.53) vs 1.93 (±0.95), the level of hemoglobin (g/L) had an average of 127.7 (±21.18) vs 133.32 (±17.36), the level of platelets ( /L) was 261.08 (±121.09) vs 245.65 (±92.97), the cell count of the level of D-dimers (mg/mL) was 7.71 (±89.52) vs 0.76 (±0.13), the level of blood sugar level (Glu g/L) had an average of 1.98 (±1.08) vs 1.41 (±0.83), the level of glycated hemoglobin HbA1C (%) had an average of 8.21 (±2.43) vs 6.57 (±3.50), the levels of alanine aminotransferase (U/L) had an average of 45.18 (±48.61) vs 32.94 (±25.34), the levels of aspartate aminotransferase (U/L) had an average of 55.41 (±53.80) vs 35.20 (±35.16), the level of potassium (mmol/L) had an average of 4.43 (±1.20) vs 4.13 (±0.62), the level of sodium (mmol/L) had an average of 136.46 (±11.51) vs 131.24 (±30.41), the level of urea (g/L) had an average of 0.85 (±2.87) vs 0.59 (±3.26), the level of creatinine (mg/L) had an average of 15.72 (±19.67) vs 11.64 (±12.23), the level of troponin I (ng/mL) had an average of 845.37 (±7643.66) vs 267.19 (±996.36), the level of lactate dehydrogenase (U/L) had an average of 565.08 (±304.56) vs 226.92 (±113.86), the level of C-reactive protein (mg/L) had an average of 152.45 (±126.10) vs 35.63 (±52.08), and the ferritin level (ng/mL) had an average of 730.49 (±530.46) vs 531.27 (±699.75).

Evolution

During evolution, patients experienced multiple complications, the most relevant ones are described in Table 2.

**Table 2:** Main complications and biological abnormalities according to severity. *Values represented in this table are numbers (frequencies %) except where it is indicated otherwise. *The frequencies listed in this table represent the proportion of patients with each finding among those tested or assessed for the finding. Not all patients were tested or assessed for each. * bRR, brut relative risk; LDH, lactate dehydrogenase *p values state differences between non-severe and severe patients

COVID-19 patients				
Variables	Non-severe	Severe	p-value	_b_RR
Superinfection (n=915)	51 (12.4%)	360 (87.6%)	<0.001	9.80
Anemia (n=713)	48 (16.6%)	241 (83.4%)	<0.001	2.37
Leucopenia (n=691)	23 (33.3%)	46 (66.7%)	0.174	
Lymphopenia (n=579)	16 (5.5%)	273 (94.5%)	<0.001	15.28
Leukocytosis (n=691)	20 (7.4%)	250 (92.6%)	<0.001	7.90
Hyperglycemia (n=433)	38 (11.3%)	298 (88.7%)	<0.001	3.68
Thrombocytopenia (n=672)	19 (19.4%)	79 (80.6%)	0.112	1.54
Mildly elevated liver enzymes (n=413)	11 (8.5%)	119 (91.5%)	<0.001	3.29
Acute renal failure (n=652)	37 (19.6%)	152 (80.4%)	0.036	1.55
Inflammatory syndrome (n=621)	85 (15.7%)	457 (84.3%)	<0.001	8.78
Elevated D-dimers (> 1 mg/mL) (n=401)	14 (7.1%)	184 (92.9%)	<0.001	6.62
Elevated LDH (n=204)	5 (2.7%)	178 (97.3%)	NA	32.36

Among the severe patient’s group, 20.3% of patients (116) had received invasive ventilation and 40.3% (230) had been admitted to the resuscitation unit, 258 (45.2%) patients deceased during hospitalization, and 313 (54.8%) were discharged alive. Of the latter, 112 were discharged with supportive care (19.6%), 15 (2.6%) were discharged against medical advice, and 2 (0.4%) were transferred to a different facility. Throughout the 30 days of follow-up, 16 deaths were added to the total number of deaths raising the death rate to 48.0% (274) and 15 (2.6%) were lost to follow-up. No deaths were reported among the non-severe patient’s group. Table 3 shows the main characteristics among survivors and non-survivors in the severe patient’s group.

**Table 3:** Characteristics of survivors among severe patient’s group. *Values represented in this table are numbers (frequencies %) except where it is indicated otherwise *LMWH, low molecular weight heparin; bRR, brut relative risk *p values states differences between non-severe and severe patients

COVID-19 severe patients (n=571)				
Characteristics	Non-survivor (n=274)	Survivor (n=297)	p-value	_b_RR
Age subgroups (n=571)			<0.001	
18-49	6 (19.4%)	25 (80.6%)		
40-64	102 (39.4%)	157 (39.4%)		
>65	166 (59.1%)	115 (40.9%)		
Gender (n=571)			0.013	1.54
Male	187 (51.9%)	173 (48.1%)		
Female	87 (41.2%)	124 (58.8%)		
Admission unit (n=571)			<0.001	
Intermediate care unit	21 (30.0%)	49 (70.0%)		
Intensive care unit	95 (31.1%)	210 (68.9%)		
Resuscitation unit	158 (80.6%)	38 (19.4%)		
Transfer to resuscitation unit (n=571)	25 (73.5%)	9 (26.5%)	0.002	3.21
Hospital stay [mean(±SD)] (n=571)	6.89 (±5.89)	7.28 (±5.75)	0.111	
Invasive ventilation (n=571)	109 (53.2%)	7 (6.0%)	<0.001	27.37
Respiratory distress syndrome during hospitalization (n=352)	100 (79.4%)	88 (46.8%)	<0.001	6.031
Pulmonary embolism (n=571)	27 (38.6%)	43 (61.4%)	0.092	
Elevated D-dimers (>1 mg/mL) (n=319)	90 (68.1%)	94 (48.1%)	0.001	2.16
Glycemic imbalance (n=347)	89 (47.3%)	80 (52.7%)	0.418	
Lymphopenia (n=426)	165 (60.4%)	108 (39.6%)	<0.001	2.50
Acute kidney failure (n=488)	91 (59.9%)	61 (40.1%)	0.001	1.89
Mildly elevated liver enzymes (n=336)	66 (55.5%)	53 (44.5%)	0.017	1.72
Lung involvement (n=571)			<0.001	
Minor to moderate <25%	8 (21.6%)	29 (78.4%)		
Extended 25%-50%	62 (39.2%)	96 (57.6%)		
Severe 50%-70%	95 (42.4%)	129 (38.2%)		
Critical > 75%	103 (70.5%)	43 (29.5%)		
Superinfection (n=571)	169 (46.9%)	191 (53.1%)	0.515	
Inflammatory syndrome (n=487)	223 (48.8%)	234 (51.2%)	0.058	
LMWH intake (n=571)	249 (46.8%)	283 (53.2%)	0.037	0.49
Hydroxychloroquine intake (n=565)	139 (43.8%)	178 (56.2%)	0.018	0.66
Azithromycin intake (n=571)	267 (54.4%)	224 (45.6%)	0.005	0.50
Azithromycin intake before admission (n=564)	49 (38.3%)	79 (61.7%)	0.015	0.61
Antibiotics used before admission (n=564)	62 (39.5%)	95 (60.5%)	0.015	0.63

Multivariate analysis

Table 4 reports the binary logistic regression model used to adjust on confounding factors; we adjusted on sex, age, high blood pressure, BMI, and variables that were significantly associated on univariate analysis, including variables with p<0.20. The variable of interest was severity, using the code 0 for non-severe forms and 1 for severe forms. The severity was significantly associated with male gender with an aOR=2.91, 95% confidence interval {CI}: 1.42-5.94, age over 65 years old with an aOR=2.68, 95% CI: 1.25-5.73 (p<0.05), diabetes (aOR=2.18, 95% CI: 1.08-4.39), elevated D-dimers (>1 mg/mL) (aOR=6.09, 95% CI: 2.80-13.23), superinfection (aOR=3.78, 95% CI: 1.88-7.59), and baseline lymphopenia (<1000 c/mm3) (aOR=8.66, 95% CI: 3.47-21.58).

**Table 4:** Binary logistic regression model for the relationship between COVID-19 severity and risk factors. *aOR, adjusted odds ratio; 95% CI, 95% confidence interval *p values indicate differences between non-severe and severe patients

Variables	aOR	95% CI	p-value
Gender			0.003
Female	1		
Male	2.91	1.42-5.94	
Age			0.001
< 65	1		
≥ 65	2.68	1.25-5.73	
Diabetes	2.18	1.08-4.39	0.030
Elevated D-dimers	6.09	2.80-13.23	<0.001
Superinfection	3.78	1.88-7.59	<0.001
Baseline lymphopenia	8.66	3.47-21.58	<0.001

## Discussion

This study examined the relationship between the severity of confirmed COVID-19 cases in Tangier, Morocco and their clinical, biological and imaging features, from September 2020 to October 2021.

Our findings have revealed significant differences between the non-severe patient’s group and the severe patient’s group. Consistently with previous researches, our results showed that the non-severe group has less comorbidities, less chronic use of medication, a shorter symptoms-diagnosis window, and had started antibiotic therapy prior to admission. After adjusting on confounding factors, we found that male gender, older age, diabetes, elevated D-dimers, superinfection, and baseline lymphopenia were independent and significant predictors of a higher risk of disease severity.

Studies so far have shown that COVID-19 disease outcomes are not the same in all population groups. [13]. Age is noted to be the strongest risk factor for COVID-19 disease severity [13], results from the IMPACC study, phenotyping disease severity in a hospitalized COVID-19 cohort, found that age ≥ 65 years was associated with more severe disease evolution and poor prognosis [14].

Similar to other cohorts, our results showed that males were more likely to be infected with SARS‑CoV‑2 than females with a sex ratio M/F of 1.28 and more at risk to develop severe forms (OR = 2.91). These results are consistent with what the Chinese Center for Disease Control and Prevention Team (China CDC) communicated in their report in 2020 [15]; patients who needed invasive ventilation were more likely to have older age and to be from male gender. In a recent study assessing the role of androgens in severity and mortality rates of COVID-19 [16]. Ataei et al. found that androgen-mediation mechanisms could explain this sex difference, the androgens increase the expression of type II transmembrane serine protease (TMPRSS2) and angiotensin converting enzyme 2 (ACE2), these two enzymes enables new corona virus entry into host cell and their expression is higher in young males than females [16].

Early observational data suggested diabetes to be a risk factor of COVID-19 severity and mortality as we observed that diabetes patients are susceptible to develop a severe form of the disease [17]. In March 2020, Fadini et al. published a meta-analysis about prevalence and impact of diabetes among people infected with SARS‑CoV‑2, they retrieved 12 studies reporting data from 2108 Chinese patients with confirmed SARS‑CoV‑2 infection. Six of the studies analyzed found that the pooled risk ratio (RR) of diabetes among patients with severe infection was 2.26 (95% CI 1.47-3.49) compared to those with less severe infection. Based on this meta-analysis, they concluded that diabetes may worsen the outcome of SARS-CoV-2 infection [18] consistently with the excess mortality due to infectious diseases in diabetic patients [19]. Another meta-analysis among Chinese COVID-19 patients reported that diabetes was associated with a higher risk of COVID-19 severity or death (RR: 2.96; 95% CI: 2.31−3.79), in this work, Guo et al. included nine studies from different provinces/cities in China combining 1070 patients with diabetes from 8807 patients. They found a significant association of pre-existing diabetes with disease severity or death [20]. Patients with type 2 diabetes are characterized by an increased expression of ACE2 in the lungs [21], which is caused by chronic inflammation, endothelial cell activation, and insulin resistance that exacerbates the inflammatory response and leads to alveolar-capillary barrier dysfunction and could explain the severe COVID-19 prognosis in diabetic patients [22].

Our results are consistent with scientific literature evidence, in fact, among the earliest researchers, Guan et al. studied Clinical Characteristics of Coronavirus Disease 2019 in China and found that patients with severe disease had more laboratory abnormalities including lymphocytopenia and leukopenia, than those with non-severe disease and lymphocytopenia was present in 83.2% of the patients on admission [23]. Also, a constant decrease in the lymphocyte count was described to be an early indicator of progression to severity in COVID-19 patients [24]. The IMPACC study also pointed out that risk factors associated with prolonged hospitalization included baseline lymphopenia (OR 2.19; 95% CI 1.61-2.97) [14]. Zhang et al. presented an AI-based system, clinically applicable AI system for accurate diagnosis, quantitative measurements, and prognosis of COVID-19 pneumonia using CT that can help to predict the prognosis of COVID-19 patients. In this study, D-dimer levels were significantly higher in severe COVID-19 patients compared to non-severe patients [25]. A meta-analysis combining 5872 COVID-19 patients stated that in these patients, disease severity and mortality were associated with higher D-dimer levels [26]. A review described the risk factors for severity in COVID-19 patients and found that lymphopenia and elevated levels of D-dimer are major risk factors of severe clinical course in COVID-19 patients [24]. In addition to that, the clinical characteristics of 138 hospitalized patients with 2019 novel coronavirus-infected pneumonia in Wuhan, China study [27] reported that in non-survivors, they observed decline in the lymphocyte count and rise in the D-dimer over time in contrast with more stable levels in survivors. And while studying post-COVID syndrome (PCS) in Germany, a prospective, multi-center, population-based cohort study looking at severity, predictors, and clinical correlates of post-COVID syndrome, also revealed significant differences between participants with high and low PCS score regarding markers of thrombosis (D-dimer) [28].

Varying rates of co-infections and/or superinfections in patients with COVID-19 infections were described in the literature. And even if a distinction between the two was often not clear, these infections appear to be associated with severity of COVID-19 and poor outcomes. In our study we found that superinfection was associated with a higher risk of disease severity. This could be explained by the fact that a viral infection predisposes the host to a bacterial pneumonia considered to lead to more severe symptoms and eventually worsen the disease outcomes and the prognosis is usually poor. Studies that have investigated the risk and impact of coinfection and superinfection on patients with COVID-19 have shown conflicting results; three distinctive associations have been reported, improved, deteriorated, and no effect. A literature review was conducted by Omoush and Alzyoud to provide updated evidence regarding the prevalence and impact of coinfection and superinfection on the severity and outcome of COVID-19 infection and the possible mechanisms underlying the higher risk of coinfection and superinfection in SARS-CoV-2 patients [29]. Their results have shown a variability concerning the rate of coinfection and superinfection in patients with SARS-CoV-2; but their risk was found to be considerable. Particularly, bacterial and viral coinfection/superinfection seem to have negative consequences on the outcomes of COVID-19 patients and significantly increase the risk of mortality and critical illness. However, the impact of a parasitic coinfection on the outcomes of COVID-19 patients was found unclear.

In our study, we did not succeed to associate smoking with increased risks of disease severity like in other studies, [27], however, it is essential to mention that history of smoking could be under reported in our cohort like the Zhang and colleague’s study [25] where 7% of the investigated population was identified as smokers contrasting with smoking prevalence among Chinese men that was known to be over 60% [30].

To the best of our knowledge, this is one of the first studies to investigate the relationship between risk factors and COVID-19 severity in Morocco. One of the strong aspects of our study remains in the design we employed, the use of longitudinal studies is an approach that allowed us to describe the disease severity and to improve our understanding of the patient's evolution. We also combined prospective and retrospective methods; the prospective part helped reducing missing data generated by the retrospective phase. Also, we estimated that the inclusion timeframe was long enough to provide representativity over time.

Not to mention that our study used the ISARIC-WHO CRF (modified), which standardized data collection for all patients included in the study, eliminating information bias and allowing comparability of data with other studies and countries.

This study was conducted in a single center; however, Tangier is considered to be an area of summering and of high multiethnic Moroccan frequentation welcoming the inhabitants of the different regions of the country, which makes the recruited sample representative of the Moroccan population and so results may be generalizable. But for validation, multiple prospective studies still need to be carried out. Furthermore, the retrospective part of the study lacks control of variables, we have attempted to control for confounding factors, but the possibility of unmeasured or residual confounders still exists.

To better assist in quickly and efficiently classifying COVID-19 patients we need to use prognostic models. In the currently available models, comorbidities, sex, C reactive protein, and creatinine were identified to be prognostic factors [13], we do hope to have provided a basis for future studies to help develop more elaborated models.

It is also worth mentioning that findings from our study may serve for health economic analyses and research in order to assist public health decisions in the future.

## Conclusions

Our study showed significant associations between severity of COVID-19 cases and age over 65 years, male gender, diabetes, elevated D-dimers, lymphopenia, and superinfection. Consistent with numerous other studies, our findings will allow public health authorities to plan and build a sustainable, reactive and relevant epidemiological surveillance system and should be integrated into health emergency preparedness and response strategies ensuring a resilient health system. Unraveling the determinants of COVID-19 severity would allow to stratify population according to risk, in sort that high-risk groups would be prioritized in terms of vaccination and hospitalization. A better insight of the underlying biological mechanisms could also represent a guide for tailored therapies. Data generation concerning COVID-19 is never too much. Lessons are always to be learned from the past for future epidemic preparedness and response and we hope that our study will contribute to the scientific literature concerning this disease.

## Appendices

**Table 5:** Biological threshold used in defining biological abnormalities. *CRP, C reactive protein; ESR, erythrocyte sedimentation rate; PCT, procalcitonin; AST, aspartate transaminase; ALT, alanine transaminase; LDH, lactate dehydrogenase

Abnormality	Threshold definition
Elevated D-dimers	>1 mg/mL
Elevated CRP	>10 mg/L
Elevated ESR	females > (age×10)/2; males > age/2
Elevated ferritin	females > 200 ng/mL; males > 300 ng/mL
Elevated PCT	>2 ng/mL
Mildly elevated AST	>60 U/L
Mildly elevated ALT	>60 U/L
Elevated LDH	>245 U/L
Lymphopenia	<1 × 10^9^/L)
Elevated creatinine	females >12 mg/L ; males > 13 mg/L
Elevated urea	> 0.45 g/L
